# Association of physical fitness with health-related quality of life in early postmenopause

**DOI:** 10.1007/s11136-016-1294-6

**Published:** 2016-04-16

**Authors:** N. Moratalla-Cecilia, A. Soriano-Maldonado, P. Ruiz-Cabello, M. M. Fernández, E. Gregorio-Arenas, P. Aranda, V. A. Aparicio

**Affiliations:** 1 Department of Physiology, Faculty of Pharmacy, Faculty of Sport Sciences, and Institute of Nutrition and Food Technology, University of Granada, Granada, Spain; 2 Pinos Puente Clinical Management Unit, Granada, Spain; 3 Department of Physical Education and Sport, Faculty of Sport Sciences, University of Granada, Granada, Spain; 4 Department of Public and Occupational Health, EMGO+ Institute for Health and Care Research, VU University Medical Centre, Amsterdam, The Netherlands

**Keywords:** Functional capacity, Fitness testing, Physical health, Mental health, Flexibility, Strength, Women

## Abstract

**Objective:**

To assess the association of different components of physical fitness with HRQoL in early postmenopause and to test which physical fitness components are independently associated with the physical and mental components of HRQoL.

**Methods:**

The final sample comprised 67 early postmenopausal women. Physical fitness was assessed with the Senior Fitness Test battery (additionally including handgrip strength test), and HRQoL was evaluated with the Short-Form Health Survey-36 (SF-36). We also analyzed plasma gonadotropic hormones and estradiol.

**Results:**

Overall, most of the fitness components were positively associated with HRQoL. Lower-body flexibility, upper-body muscle strength and cardiorespiratory fitness were the fitness components more strongly associated with HRQoL (*r* range from 0.28 to 0.56). Static balance was especially associated with mental health (*r* = −0.46, *P* < 0.001). Lower-body flexibility (assessed with the chair sit-and-reach test) and upper-body muscle strength (assessed with handgrip dynamometry) were independently associated with the SF-36 Physical Component Summary (both, *P* < 0.001). Upper-body muscle strength (*P* < 0.01) and cardiorespiratory fitness (assessed with the 6-min walk test, *P* < 0.05) were independently associated with the SF-36 Mental Component Summary.

**Conclusions:**

Higher physical fitness is associated with better HRQoL in early postmenopause. Lower-body flexibility and upper-body muscle strength were the most important independent fitness indicators, explaining ~30 % of HRQoL.

## Introduction

The physiological impact of menopause on women’s health has been deeply explored. Menopause increases cardiometabolic risk factors due to the significant decline in the estrogen levels and the testosterone predominance [1]. Consequently, postmenopause appears to be associated with higher incidence of metabolic syndrome and cardiovascular diseases [2–4]. These hormonal changes occurring in women during and after menopause transition may also have a relevant impact on health-related quality of life (HRQoL) [5, 6], especially at the psychological, physical and sexual spheres [7, 8]. Indeed, the postmenopausal period is also characterized by higher incidence of depressive symptoms [9]. Furthermore, early postmenopause is a relevant period in women’s life that is characterized by even a greater prevalence of vasomotor menopausal symptoms such as hot flushes, which negatively impact their HRQoL in comparison with pre-menopausal women [6].

Higher levels of physical activity are closely related to higher physical fitness [10, 11], a more favorable cardiometabolic profile [12, 13], lower levels of depression [14], lower frequency of hot flushes [15] and better HRQoL [16–18] in postmenopausal women. However, the association of different components of physical fitness with HRQoL in early postmenopause is not well documented. Since physical fitness is a multicomponent modifiable factor strongly related to health and disease in different populations [19, 20], and early postmenopause is a rather critical period in women’s health, it is important to comprehensively characterize the association of different fitness components with HRQoL in this specific population. In addition, it is also of clinical interest to address the extent to which different components of fitness (cardiorespiratory fitness, muscle strength, flexibility and balance/motor agility) are independently related to mental and physical HRQoL, which might have important implications for the development and implementation of future exercise-based intervention programs in early postmenopausal women.

The aims of the present study were: (1) to assess the association of different components of physical fitness with HRQoL in early postmenopausal women and (2) to examine which fitness components are independently related to HRQoL in this specific population.

## Methods

### Participants and study design

A descriptive, cross-sectional study using a convenience sample was conducted. The recruitment of participants was performed by researchers and staff from primary care centers from Granada, southeast Spain, via information panels, lectures, e-mails, letters or telephone. We recruited 198 women. All participants were informed about the aims and study procedures and signed written informed consent before taking part in the study. The general inclusion criteria were: (1) to be between 40 and 65 years old; (2) not to have acute or terminal illness or severe cognitive impairment (a score <10 in the Mini-Mental State Evaluation [MMSE] [21]); (3) lack of neuromuscular disease or drugs affecting neuromuscular function; (4) to be able to ambulate without assistance; (5) not to have suffered major cardiovascular event in the past (i.e., myocardial infarction, angina or ictus). The study was reviewed and approved by the Ethics Committee of the “Hospital Virgen de las Nieves” (Granada, Spain).

### Procedures

Measurements were performed on two alternate days (e.g., Monday and Wednesday) by the same trained group of researchers. On day 1, the MMSE was performed by personal interview in a private room. Thereafter, socio-demographic (self-reported questionnaire including age, educational status and current occupational status) and clinical data were obtained. Subsequently, biochemical analysis and anthropometric and body composition parameters were assessed. Participants then received the *Short*-*Form Health Survey-36* (SF-36) [22] to be filled at home (middle day). On day 2, the research team verified that the questionnaire was completely and correctly filled, and physical fitness was assessed.

#### Anthropometry and body composition

A portable eight-polar tactile-electrode bioimpedance device (InBody R20, Biospace, Seoul, Korea) was used to measure body fat percentage and muscle mass. Height (cm) was measured using a stadiometer (Seca 22, Hamburg, Germany). Body mass index (BMI) was calculated (weight[Kg]/height[m^2^]).

#### Hormonal assessments

Venous blood samples after all night fasting were collected. Immediately after the blood collections, the samples were brought to the hospital biochemical analysis laboratory, where they were centrifuged, and pipetted. Plasma luteinizing hormone (LH), follicle-stimulating hormone (FSH) and estradiol hormone concentrations were estimated using an autoanalyzer (Hitachi-Roche p800, F. Hoffmann-La Roche Ltd. Switzerland).

#### Health-related quality of life

We assessed HRQoL with the SF-36 [22]. The SF-36 contains 36 items grouped into 8 dimensions: physical functioning, physical role, bodily pain, general health, vitality, social functioning, emotional role and mental health. The scores range from 0 to 100 in every dimension, where higher scores indicate better health. The physical component summary (range 0–100) and the mental component summary (range 0–100) were also calculated.

#### Physical fitness

Physical fitness was assessed with the Senior Fitness Test battery [23] (6-min walk, chair-stand, chair sit-and-reach, back scratch and 8-foot up-and-go tests), additionally including the handgrip strength test [24] and the blind flamingo test [25].

### Statistical analysis

Bivariate partial correlation (adjusted for age, BMI, educational and occupational status) was used to assess the association of the different components of physical fitness with the eight dimensions of HRQoL, as well as with the physical and mental component summary of the SF-36 (objective 1). To test which fitness component were independently associated with HRQoL (objective 2), a forward stepwise regression analysis was carried out including the physical component summary and the mental component summary of the SF-36 as dependent variables in separate models. For each of the two models, age, educational and occupational status were included and kept fixed into the model (so that potential confounding was controlled). Thereafter, the eight fitness tests were simultaneously introduced into the model using a stepwise procedure. This procedure introduces the fitness tests step-by-step into the model (when *P* < 0.05) according to the strength of their association with the outcome. The model is reassessed with the addition of every new fitness test and variables are left out of the model if *P* > 0.10.

The statistical analysis was performed with SPSS (IBM SPSS Statistics for Windows, version 20.0; Armonk, NY, USA), and the statistical significance was set at *α* = 0.05.

## Results

One women was >65 years old, 36 did not complete or had valid data in all the physical fitness assessments, and 94 women did not met the criteria to be considered postmenopausal women (at least one year with amenorrhea and FSH > 20 mg/dL with a LH/FSH index < 0.7 or estradiol levels < 40 pg/mL). Therefore, the final study sample comprised 67 early postmenopausal women whose socio-demographic and clinical characteristics are given in Table 1.

**Table 1:** Descriptive characteristics of the early postmenopausal participants (*n* = 67)

Variables	Mean ± SD
Age (years)	55.6 ± 5.0
Weight (kg)	70.3 ± 11.0
Height (cm)	155.1 ± 6.0
Body mass index (kg/m^2^)	30.1 ± 5.8
Body fat (%)	40.0 ± 7.0
*Gonadotropic hormonal profile*	
LH (mg/dL)	29.3 ± 12.7
FSH (mg/dL)	69.6 ± 25.8
LH/FSH	0.42 ± 0.13
Estradiol (mg/dL)	15.9 ± 15.5
*Physical fitness*	
Cardiorespiratory fitness	
6-min walk (m)	501.1 ± 55.6
Muscle strength	
Upper body	22.7 ± 5.4
Handgrip strength (kg)	
Lower body	13.1 ± 3.3
30-s chair-stand (rep)	
Flexibility	
Upper body	
Back scratch (cm)	−6.3 ± 9.7
Lower body	
Chair sit-and-reach (cm)	−0.5 ± 13.5
Balance	
Dynamic/agility	
8-feet up-and-go (s)^*#*^	6.1 ± 1.2
Static	
Blind flamingo (fails)^#^	7.4 ± 4.2
*Health-related quality of life (SF-36)*	
Physical functioning	72.6 ± 30.9
Emotional role	76.2 ± 39.8
Physical role	60.4 ± 43.4
Vitality	55.5 ± 24.5
Mental health	62.7 ± 25.7
Social functioning	81.5 ± 25.4
Bodily pain*	54.8 ± 28.4
General health	56.9 ± 26.8
SF-36 physical component summary	60.8 ± 26.0
SF-36 mental component summary	69.0 ± 23.4
*Educational status*	*n (%)*
No studies	9 (13.4)
Primary school	45 (67.2)
Secondary school/professional training	6 (9.0)
University degree	7 (10.4)
*Occupational status*	
Home worker	26 (38.8)
Unemployed/retired	28 (41.8)
Partial or full employed	13 (19.4)

*SD* standard deviation, *BMI* body mass index, *LH* *luteinizing hormone*, *FSH* *follicle-stimulating hormone*, *SF-36* short-form-36 health survey

* Higher values indicate less pain

# Lower scores indicate better performance

Partial correlations after adjustment for age, BMI, educational and occupational status between physical fitness components and HRQoL (as assessed through the different SF-36 dimensions and standardized components; objective 1) are given in Table 2. Overall, higher levels of physical fitness were significantly associated with better HRQoL. Lower-body flexibility (*r* range from 0.31 to 0.56), upper-body muscle strength (*r* range from 0.28 to 0.48) and cardiorespiratory fitness (*r* range from 0.28 to 0.32) were the fitness components more strongly associated with HRQoL. Static balance showed a moderate association with the mental health subscale of the SF-36 (*r* = −0.46, *P* < 0.001). Physical fitness was particularly associated with the vitality and bodily pain dimensions of the SF-36 (*r* range from 0.28 to 0.56).

**Table 2:** Partial correlations of health-related quality of life with physical fitness in early postmenopausal women (*n* = 67)

SF-36 dimensions	Muscle FITNESS		Flexibility		Balance		Cardiorespiratory fitness
	Upper body	Lower body	Upper body	Lower body	Static	Dynamic	
	Handgrip strength	Chair-stand	Back scratch	Chair sit-and-reach	Blind flamingo^#^	8-feet up-and-go^#^	6-min walk
Physical functioning	0.289*	−.019	0.079	0.178	−.198	−.165	0.127
Emotional role	0.279*	0.049	0.207	0.307*	−.128	−.023	0.299*
Physical role	0.390**	0.235	0.298*	0.524***	−.130	−.161	0.190
Vitality	0.406**	0.222	0.156	0.476***	−.243	−.276*	0.313*
Mental health	0.155	0.056	0.104	0.239	−.459***	−.082	0.124
Social functioning	0.438**	0.103	0.047	0.213	−.102	−.083	0.214
Bodily pain^Ψ^	0.481***	0.205	0.324*	0.557***	−.217	−.217	0.315*
General health	0.371**	0.290*	0.114	0.356**	−.164	−.191	0.135
Physical component summary	0.475***	0.217	0.266	0.510***	−.218	−.224	0.233
Mental component summary	0.401**	0.108	0.185	0.385**	−.271	−.132	0.283*

The models were adjusted for age, body mass index, educational status and employment status

*SF-36* Short-Form Health Survey-36

* *P* < 0.05; ** *P* < 0.01; *** *P* < 0.001

^Ψ^Higher values indicate less pain

^#^Lower scores in that test indicate better performance

The independent association of different fitness components with HRQoL (physical and mental component summary of the SF-36, objective 2) is presented in Table 3. Lower-body flexibility (assessed with the chair sit-and-reach test) and upper-body muscle strength (measured with handgrip dynamometry) were independently associated with the SF-36 physical component summary (both, *P* < 0.001). The final model explained 35 % of the variability of the physical component (*P* < 0.001). Upper-body muscle strength (*P* < 0.01) and cardiorespiratory fitness (assessed with the 6-min walk test, *P* < 0.05) were independently associated with the SF-36 mental component summary. The final model explained 27 % of the variability in mental health (*P* < 0.01).

**Table 3:** Stepwise regression analysis assessing which fitness components were independently associated with health-related quality of life in early postmenopausal women

	β	B	SE	*P*	*R* ^2^	*R* ^2^ change	*P*
*SF-36 physical component summary*							
Step 1					0.032		0.573
Age	0.145	0.742	0.649	0.258			
Educational status	0.126	3.040	3.200	0.345			
Occupational status	−0.038	−0.909	3.129	0.773			
Step 2					0.290	0.258	<0.001
Age	0.152	0.777	0.561	0.172			
Educational status	−0.064	−0.002	2.838	0.982			
Occupational status	0.004	0.096	2.711	0.972			
Chair sit-and-reach	0.523	0.988	0.212	<0.001			
Step 3					0.349	0.059	<0.001
Age	0.151	0.773	0.542	0.159			
Educational status	0.001	0.027	2.741	0.992			
Occupational status	−0.020	−0.474	2.630	0.858			
Chair sit-and-reach	0.377	0.713	0.236	0.004			
Handgrip strength	0.283	−13.313	0.598	0.025			
*SF-36 mental component summary*							
Step 1					0.275		0.643
Age	0.108	0.480	0.480	0.401			
Educational status	0.129	2.704	2.794	0.337			
Occupational status	−0.091	2.737	−1.885	0.493			
Step 2					0.194	0.167	0.011
Age	0.109	0.488	0.522	0.350			
Educational status	0.082	1.724	2.580	0.507			
Occupational status	−0.109	−2.254	2.514	0.374			
Handgrip strength	0.412	1.750	0.497	0.001			
Step 3					0.266	0.072	0.002
Age	0.165	0.739	0.513	0.155			
Educational status	−0.034	−0.719	2.685	0.790			
Occupational status	−0.059	−1.216	2.459	0.623			
Handgrip strength	0.361	1.533	0.487	0.003			
6-min walk	0.304	0.125	0.052	0.020			

*SF-36* Short-Form Health Survey-36, *SE* standard error, *CI* confidence interval, *β* standardized regression coefficient, *B* nonstandardized regression coefficient, *R* ^*2*^ adjusted coefficient of determination, expressing the percent variability of the dependent variable explained by each model, *R* ^*2*^ change, additional percent variability explained by the model due to the inclusion of the new term

## Discussion

The main findings of the present study indicate that higher physical fitness is consistently associated with better HRQoL in early postmenopausal women, regardless of the fitness component evaluated. Lower-body flexibility (assessed with the chair sit-and-reach test), followed by upper-body muscle strength (measured with handgrip dynamometry), were the fitness components more strongly associated with HRQoL. Lower-body flexibility and upper-body muscle strength were independently associated with the SF-36 physical component summary and both explained 35 % of the variability in physical HRQoL. Upper-body muscle strength and cardiorespiratory fitness (assessed with the 6-min walk test) were independently associated with the SF-36 mental component summary and both explained 27 % of the variability in mental HRQoL.

Our data support previous findings reporting that physical fitness is positively associated with HRQoL in postmenopausal women [23, 26–29] and extends current knowledge on the association of different fitness components with specific dimensions of HRQoL in this specific population. We observed a noticeable association of lower-body flexibility (as measured with the chair sit-and-reach test) with most of the SF-36 dimensions. Moreover, the score obtained in this test was an independent indicator of the physical component of HRQoL. On the other hand, upper-body flexibility only correlated with SF-36-bodily pain. A potential explanatory hypothesis for the great associations found between lower-body flexibility and HRQoL is that lower-body flexibility is clearly associated with bodily functionality, a key factor for better reliance, assertiveness, esteem, independence, confidence feelings and, consequently, better HRQoL [30].

Another major finding of the present study was the relatively strong relationships of upper-body muscle strength (measured with the handgrip strength test) with almost all physical and mental dimensions of HRQoL. Although muscle strength has been widely linked to better physical health and longevity [31], its relationship with HRQoL has not been studied in detail. Although not consistent [32], previous studies have shown concomitant improvements in muscle strength and HRQoL following an exercise-based intervention program [33] and our results supports a potential important role for upper-body muscle strength on HRQoL in this period of women’s life.

We also found a positive and independent relationship between cardiorespiratory fitness (assessed with the 6-min walk test) and the mental component summary of the SF-36. Previous studies have demonstrated the important cardioprotective effect of cardiorespiratory fitness in midlife women [31, 34]. Notwithstanding, no prior study has analyzed the relationship of this test with mental health components of HRQoL in postmenopause, which warrants further research.

Postmenopausal women usually experience higher anxiety and depressive symptoms [9, 35], so it is important to highlight that static balance showed a marked association with the SF-36-mental health dimension and with the SF-36 mental component summary. Balance requires concentration as well as mental equilibrium, which could partially explain this strong relationship. Moreover, the early postmenopausal period is also characterized by decreases in attention/working memory, verbal learning, verbal memory and fine motor speed [36]. Indeed, not only cardiorespiratory fitness and muscle strength but also motor fitness including movement speed, balance, motor coordination and flexibility is associated with cognitive functioning and brain activation patterns [37]. Hence, these data may suggest that exercise programs focused on enhancing flexibility, motor agility and balance in early postmenopause are mandatory in order to concurrently improve the physical and mental components of HRQoL. Indeed, exercise modalities such as yoga or Tai-Chi, where flexibility and balance activities are performed simultaneously, have shown beneficial effects on HRQoL [38–41].

This study has limitations. The sample size was relatively small and the cross-sectional design does not allow establishing causal relationships. However, physical fitness has been consistently associated with health-related benefits in different populations and it is likely that enhancing fitness might induce improvements in HRQoL. On the other hand, we contrasted the postmenopausal status through hormonal assessments, and we focused on the early stage of postmenopause, which constitutes a complicated physical and psychological period of women’s life [6]. Therefore, our results might have implications for the development of exercise-based intervention studies in postmenopausal women, with special focus on enhancing specific fitness components.

## Conclusions

The findings of the present study indicate that physical fitness is directly and consistently associated with mental and physical dimensions of HRQoL in early postmenopausal women. Lower-body flexibility and upper-body muscle strength were independently associated with the SF-36 physical component summary and both explained ~35 % of the variability in physical HRQoL. Upper-body muscle strength and cardiorespiratory fitness were independently associated with the SF-36 mental component summary and both explained ~27 % of the variability in mental HRQoL. The results from the present study could facilitate the improvement of exercise-based preventive and treatment strategies to improve HRQoL in the early stage following the menopause transition.
